# Multi-scale study of load-bearing mechanism of uplift piles based on model tests and numerical simulations

**DOI:** 10.1038/s41598-023-33221-z

**Published:** 2023-04-19

**Authors:** Jianping Fang, Songchao Lin, Kai Liu

**Affiliations:** 1 Quzhou Traffic Design Co., Ltd, Quzhou, Zhejiang People’s Republic of China; 2 Department of Civil Engineering, School of Mechanics and Engineering Science, Shanghai University, 99 Shangda Road, Shanghai, 200444 People’s Republic of China

**Keywords:** Engineering, Civil engineering

## Abstract

The uplift pile is an anti-uplift measure in engineering widely used in practice. In order to study the mechanical parameters of the pile and the surrounding soil under the uplift load, a pile uplift model test and relevant numerical test were conducted. Image analysis technique was applied to the model test to investigate the soil displacements caused by pulling the pile. The load–displacement and pile axial force-lateral friction resistance relationships were investigated at three burial depths. Comparing the model test and numerical test results, it reveals that the pile primarily underwent four stages under the influence of uplift load: initial stage of loading, strain-hardening stage, peak of loading stage, and the strain-softening stage; the soil displacements around the pile exhibited inverted conical shape as the uplift load increases; and obvious soil arching effects could be observed near the ground surface. In addition, the development of force chains and major principal stresses indicated that the pile lateral frictional resistance first increased to its maximum value and then decreased sharply along the depth direction.

## Introduction

As an effective anti-uplift measure in underground engineering, the uplift pile has been extensively utilized in the anti-uplift design of underground structures such as basements, roadways, and tunnels. Compared with the foundation bearing capacity improved by treating the soil layer, the support system formed by the anti-uplift pile and anti-uplift plate will bring a better anti-uplift effect. Wen^1^ found by the test that the anti-uplift bearing capacity of the pile under this support system was improved so that the vertical uplift of the pile was reduced by 64.2% compared with the reinforced ground method. Alawneh et al.^2^ analyzed the pile uplift resistant capacity by indoor model test to determine its main influencing factors, including pile material, type, roughness, section shape, and soil properties.

Usually, the application of pile depends on the inherent nature of the pile, including material properties, strength, and stiffness. One example is that submerged pipe infill piles are mostly used in clay, silt or silty soil, sandy soil, and artificial fill layer; and another example is screw piles are mostly used in clay, powder, sandy soil, and gravel type of strata. Meanwhile, the external factors, such as the burial depth of the pile and the soil environment, determine the nature of the pile-soil contact surface and the damage pattern of the soil particles. Chen et al.^3^ found three rupture surfaces of the damage morphology of the pullout-resistant pile, and the shape of the rupture surface determines the ultimate bearing capacity of the pullout-resistant pile and listed the equation of the rupture surface of the soil around the pile. Amjad et al.^4^ tested the pullout resistance of equal-section piles under vertical load and found that the higher the fracture rate of the soil around the pile under the same load, the lower the bearing capacity, and the higher the fracture rate under ultimate load can give full play to the soil bearing capacity instead. Some researchers found that when steel pipe piles with different burial depths were pulled up, the pile axial force increased by 26% for every 20 cm increase in burial length^5^.

On the other hand, the pile must overcome the frictional resistance when being pulled up, and the pullout resistance accompanies the frictional resistance. Some studies have shown that the rougher the pile-soil interface is, the smaller the ratio between the residual bearing capacity and the ultimate bearing capacity of the pile, and the higher the conversion efficiency of the pile bearing capacity^6,7^. By comparing the monopile vertical uplift ultimate bearing capacity of test pile and engineering pile in-situ, it is found that the uplift bearing capacity of engineering pile was much larger than that of test pile, which also explained that the frictional resistance at the contact surface was not negligible^8,9^. Qin et al.^10^ conducted a static test of the vertical pullout resistance of monopile in indoor calcareous sand and quartz sand. They found that the elevation of calcareous sand around the top of the pile was small due to the "bottleneck effect" caused by the interconnection of surface particles, which strengthened the ultimate lateral frictional resistance of the upper pile section. Hussein et al.^11^ conducted pullout tests on model piles buried in loose dry sand and dense sand with different *L*/*D* ratios (*L*/*D* = 20, 25, 30) and found that under the combined effect of seismic and pullout loads, the maximum pullout capacity of piles in loose dry sand was reduced by 55.02–73.22%, while in dense sand, the maximum pullout capacity of the three models was reduced less, i.e., the friction surface area in dense sand was smaller than that in loose sand, and the increase in relative density would increase the effective stress. In addition, the frictional resistance also affects the rate of increase in pile stress. Sakr et al.^12^ conducted pullout test on model piles (with and without anchor wings) installed in dry sand with different density and found that the pullout resistance of anchor ring piles increased with the increase of relative density of sand. When the relative density of sand was 80%, the pullout resistance of anchor wings pile reached up to 2.77 times as much as the regular pile. Other researchers also found that the lateral frictional stress gradually increases with depth and the rate of stress increase gradually decreases^13^. In addition to theoretical derivation, model tests, and field tests, numerical calculation, e.g. FEM and DEM, is widely used to study the bearing capacity of uplift-resistant piles^14–16^.

Overall, the application of counter-extraction piles in various soil layers and engineering environments has received extensive research both at home and abroad. However, the analysis of the entire process of lateral friction resistance in the micro view and the comparative micro view remains unexplored. By combining DIC image technology, three sets of pullout tests with different burial depths (burial depths 300 mm, 250 mm, and 200 mm) are designed to analyze the load–displacement relationship of the pile top and the relative displacement of soil around the pile. DEM is used to simulate the force process of a pullout-resistant pile and record the force change of soil particles around the pile. Finally, the test results were compared to those of the simulation.

## Lab tests

The research included three pullout tests conducted at pile burial depths of 300 mm, 250 mm, and 200 mm. A universal machine controlled the pile top load to ensure equal loading; strain gauges assessed the pile axial force, and the Digital Image Correlation (DIC) approach calculated the soil full-field displacement. The uplift load, displacement, pile axial force distribution, and soil displacement were all calculated.

### Test materials

**Figure 1:**
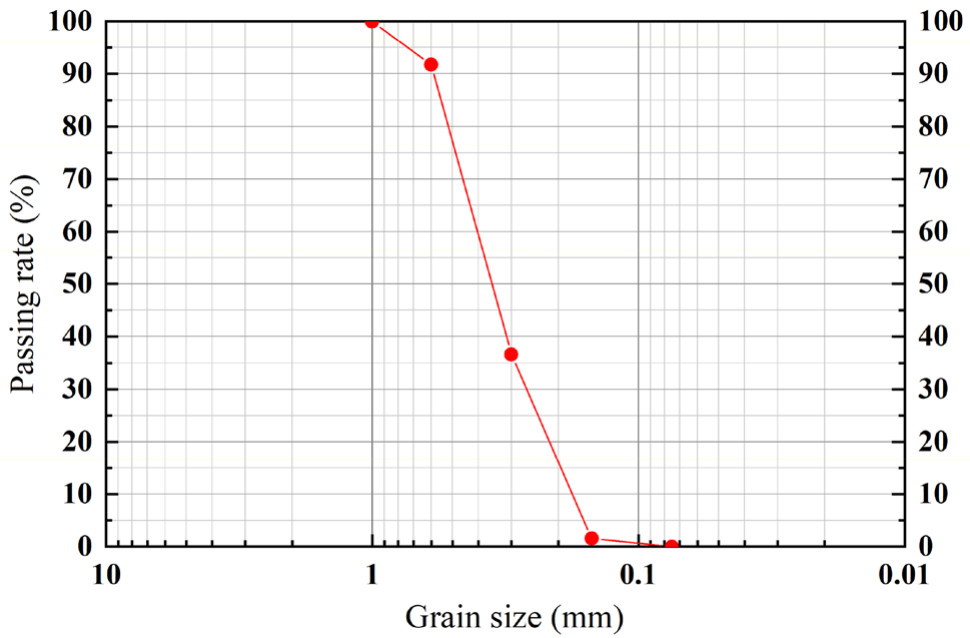
Grain size distribution curve of the tested soil.

The model pile is an aluminum thin-walled semicircular pile with an elastic modulus of 7 × 10^10^ Pa, a pile length of 300 mm, a diameter of 40 mm, and a wall thickness of 2 mm. According to Azzam et al.^17^, the model pile was applied abrasive paper externally to simulate the friction between the reinforced concrete pile and soil. The test sand is Fujian standard sand. As shown in Fig. 1, the average particle size *d*_50_ = 0.36 mm, measured by sieve test, and it is poorly graded sand according to ASTM D6913-04^18^, inhomogeneity coefficient *C*_*u*_ = 1.59, curvature coefficient *C*_*c*_ = 0.89, minimum dry density of 1.44 g/cm^3^, maximum dry density of 1.75 g/cm^3^, sand density of 1.59 g/cm^3^, relative gravity *D*_*r*_ = 0.53, internal friction angle *φ* = 30.96°, and the sand layer is in a medium dense state.

### Test setup

**Figure 2:**
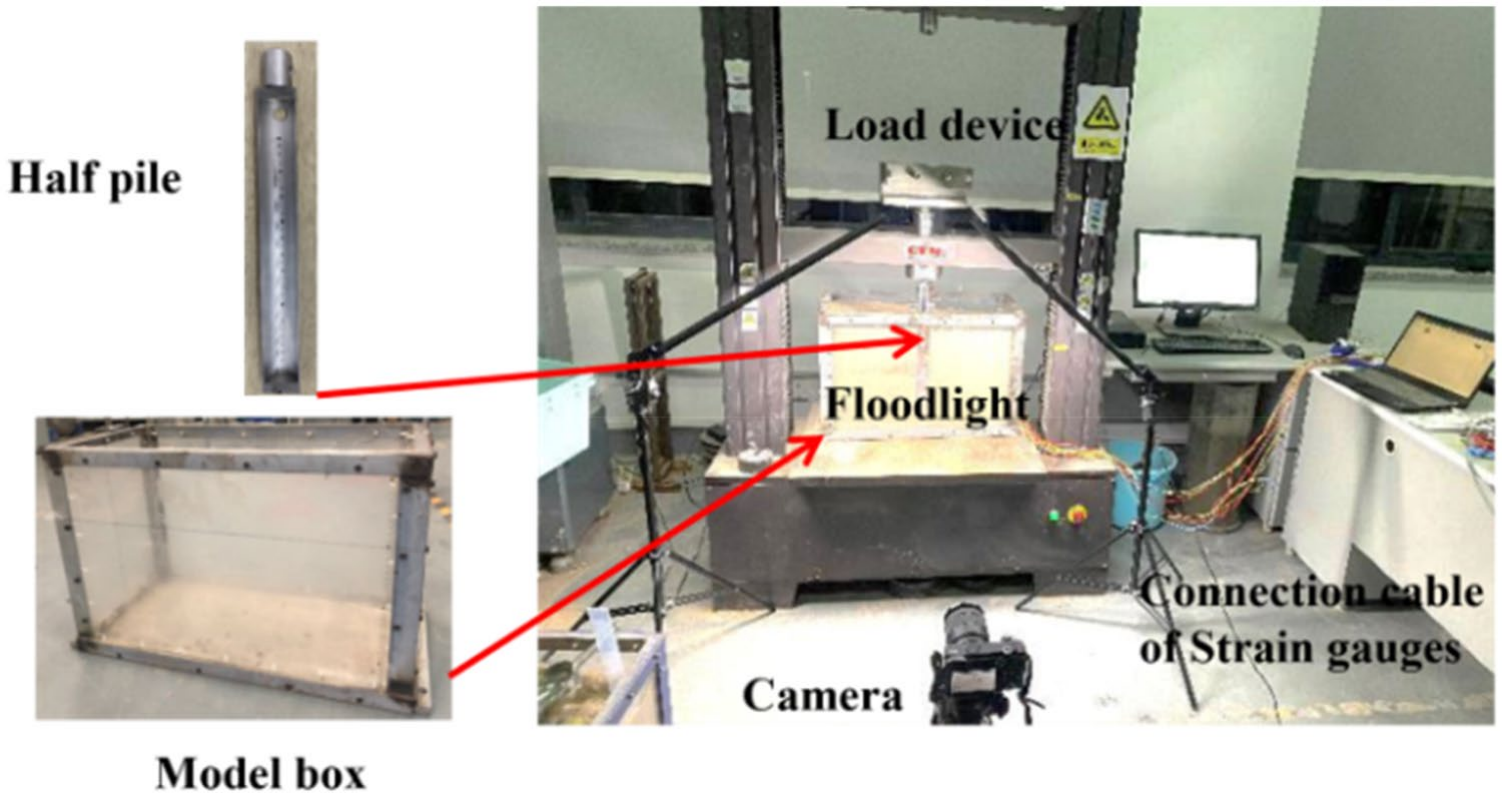
Schematic of test setup.

**Figure 3:**
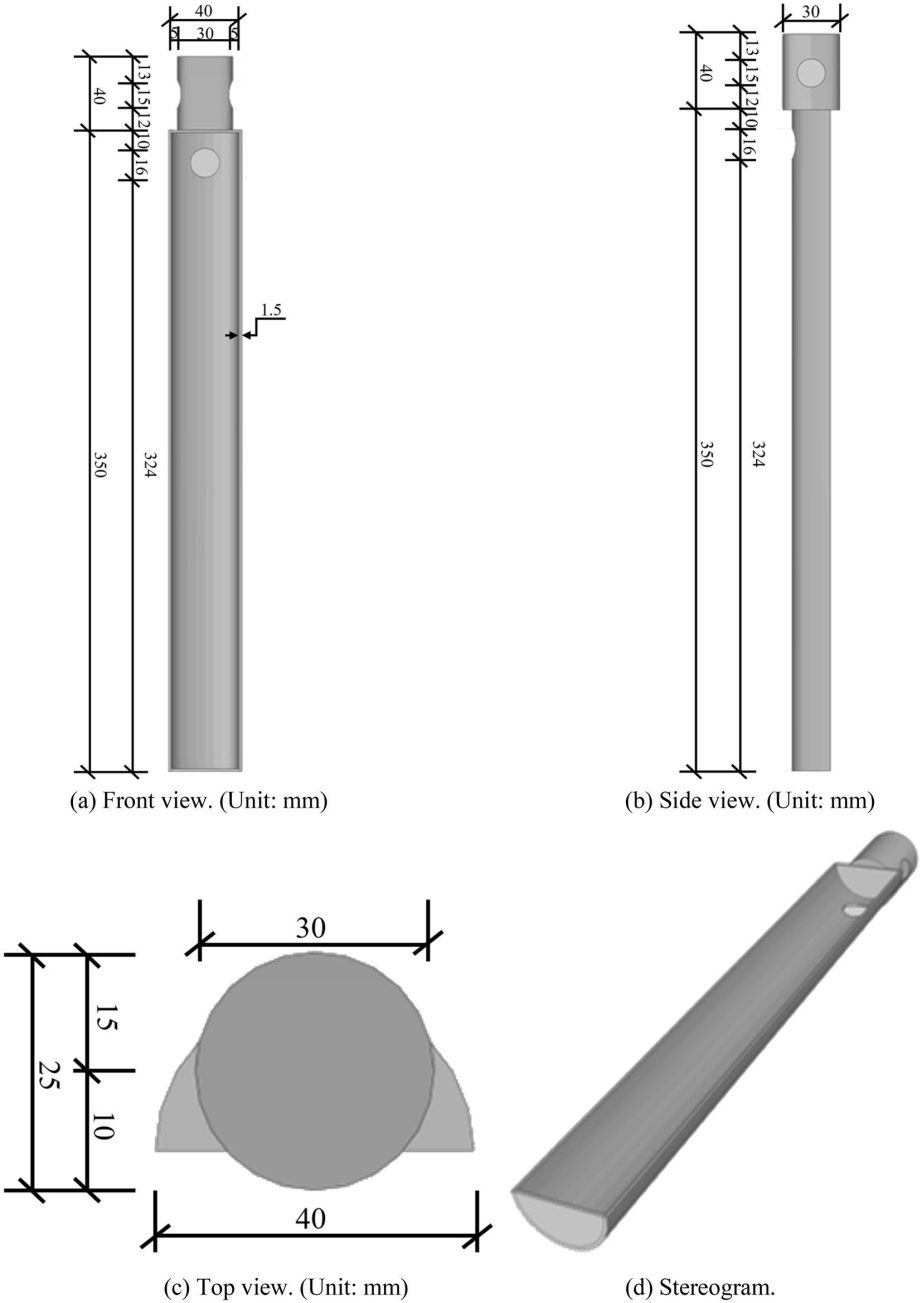
Schematics of the model pile.

The single pile studied in this paper corresponds to the pile used in the tunnel section of the Heyi River Crossing Project in Quzhou City, Zhejiang Province, China. The pile length used in the project is 10 m and the pile diameter is 1 m. To simulate the in-situ pile in the model box and eliminate the scale effect^19–22^, a reduction factor of n = 30 was applied for the reduced scale test. The test device is made up of three parts: the model box, the loading system, and the image acquisition device. The model box measures 600 mm × 290 mm × 400 mm and has four transparent sides for easy observation of the testing process. These dimensions of the model box were chosen to avoid the boundary effect. The loading device can perform equal-speed loading and has a 700 mm tensile and compressive stroke and a speed range of 0.01 mm/min to 500 mm/min. The image acquisition camera is the SONYA6500, which has a half-frame sensor with 24.2 million pixels. The system of the pile-drawing device and sketch of model pile were presented in Figs. 2 and 3, respectively.

To insure the repeatability of tests, the specimens were prepared in six layers at a height of 50 cm using the falling rain method. The production of each layer of sand went through the following steps. First, we weighed a certain mass of sand. Then the sand was hammered to a predetermined scale and leveled. Each layer of sand was hammered 20 to 30 times, and the number of hammering increased from bottom to top. According to Basha and Azzam^23^, the model pile was mainly installed by two methods: driving method and non-disturbance method. However, the pile used in this study is semi-circular pile, which is hard to control its perpendicularity during pile-driving. Therefore, the non-disturbance method was applied to install the model pile. The initial layer of sand was 10 cm thick and placed in a semi-circular pile after being laid and compacted. Subsequent layers were 5 cm thick, resulting in a total height of 35 cm. Finally, the sand was left to settle for 4 h. The digital camera was placed outside the model box, and after focusing, the image was calibrated for further image processing. Finally, the universal machine was loaded at a constant speed of 0.5 mm/min.

### DIC image technology

DIC is an optical method for measuring mechanical strain that permits non-contact measurement of the target object. In other words, two images of the target surface (a pixel is the basic unit of a digital image) are acquired at different time points, and the binary map is obtained by digitizing the images and calculating the correlation of the binary maps at the connected time points to obtain the deformation field information of the target surface.

### Analysis of lab test results

#### Analysis of pile uplift process

**Figure 4:**
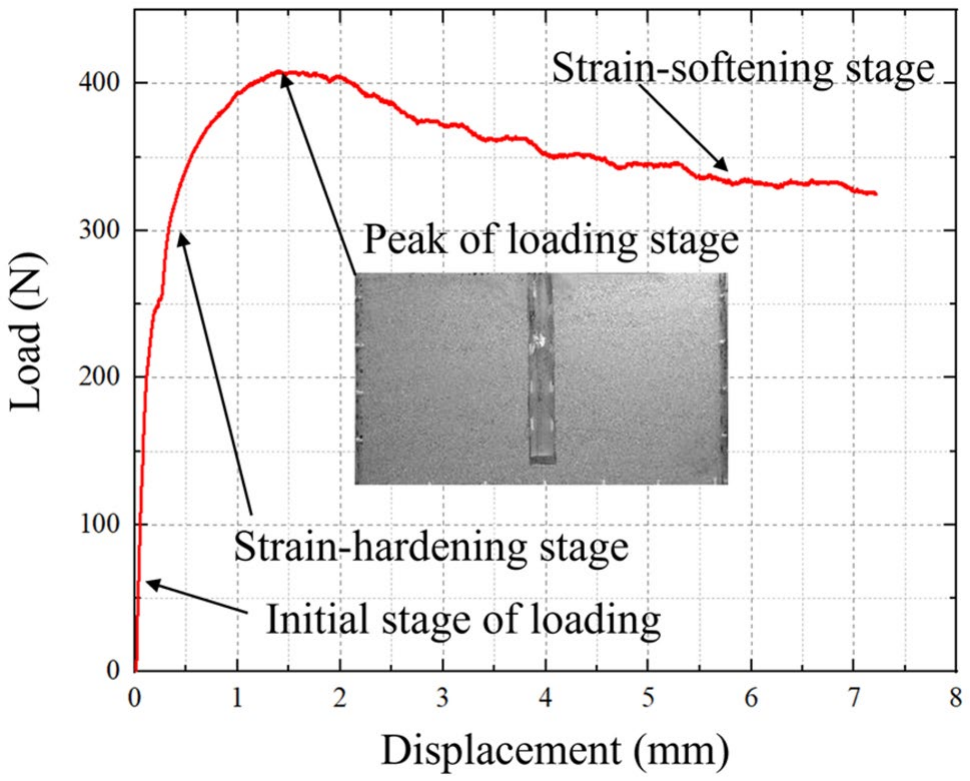
The Q-s curve of pile buried depth with 300 mm.

Using a 300 mm pile burial depth as an example, Fig. 4 depicts the load (*Q*)-displacement (*s*) curve. The first step is the initial stage of loading, in which the pile is subjected to a little pull-up force, no relative displacement occurs with the earth surrounding the pile, and the pile-soil can return to its initial position after the load is removed. The second phase is strain-hardening stage. As the pull-up force increases, the soil surrounding the pile is susceptible to increased pile-side friction and structural deterioration, which is subsequently transferred to the surrounding soil. A portion of the soil is harmed by plastic at this point. The peak of loading stage is the third phase. The sandy soil surrounding the pile has been completely harmed. Currently, the pile has reached its maximum bearing capacity, and the range of its influence on the soil has likewise reached its maximum extent. As the pile continues to pull up and the burial depth lowers, the pile bearing capacity reduces significantly with the increase in displacement during the strain-softening stage.

**Figure 5:**
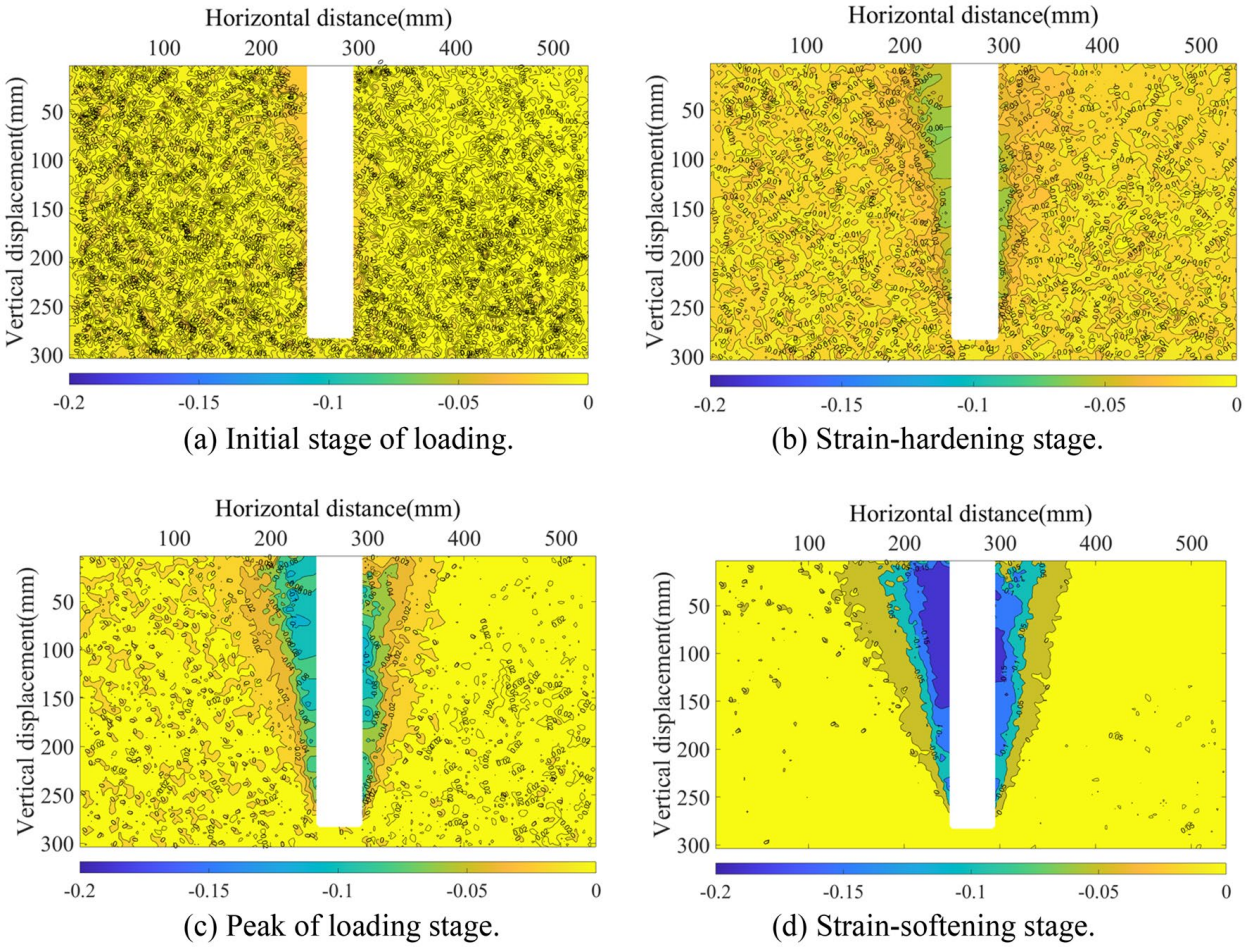
Soil displacement contour map.

When the pile is displaced by the uplift load, the surrounding earth becomes disturbed. Using the DIC non-contact image measuring technique, the displacement clouds of the four steps are acquired; refer to Fig. 5. (Soil displacement upward is negative, downward is positive). At the onset of pile uplift, the pile first induces soil displacement at the pile-soil interface, which primarily demonstrates the construction and expansion of the pile lateral frictional resistance; at this point, the effect range of the sand-soil displacement deformation is limited (Fig. 5a). As pile uplift increases, the horizontal displacement of soil surrounding the pile begins to spread outward, with the pile at its core. The disturbance range grows with the distance of pile uplift, whereas the expansion range is restricted by the mutual restraint between particles. The expansion radius is around 1.5*D* (*D* is the diameter of the model pile, Fig. 5b). The displacement cloud of the final damage is V-shaped around the pile, with the greatest concentration of soil displacement at the top of the pile. The influence range on the soil surrounding the pile grows as the pile is repeatedly raised but tends to stabilize when the soil transitions from the peak of loading stage to the strain-softening stage.

**Figure 6:**
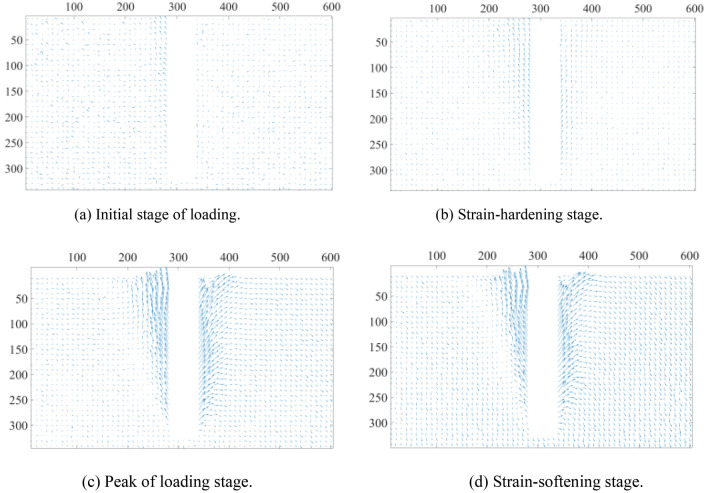
Soil displacement vector diagram.

As shown in Fig. 6, the horizontal and vertical displacements of the soil are synthesized into a vector diagram. The direction of the arrow in the diagram represents the direction of soil particle displacement, whilst the length of the arrow represents the size of the displacement. In the initial phase of uplift, the pile uplift force is always less than the lateral friction resistance, so the soil displacement vector is uniformly distributed and nearly unchanged. With the rise of the pulling force (up to the maximum lateral friction resistance), the soil movement around the pile is propelled, and it is discovered that the soil displacement in the region of the pile, above 150 mm, changes primarily in the vertical direction. At a pile depth of 300 mm, the ultimate condition of soil disturbance in the horizontal direction of approximately 100 mm surrounding the pile is extreme, and the surface is accompanied by uplift displacement. The surface uplift is quite small when the pile burial depth is relatively shallow.

#### Analysis of pile axial force and lateral frictional resistance

The strain gauges are positioned symmetrically on the pile to determine its axial force. The strain values at various points under each load level are recorded, and the corresponding cross-sectional axial force is computed.

**Figure 7:**
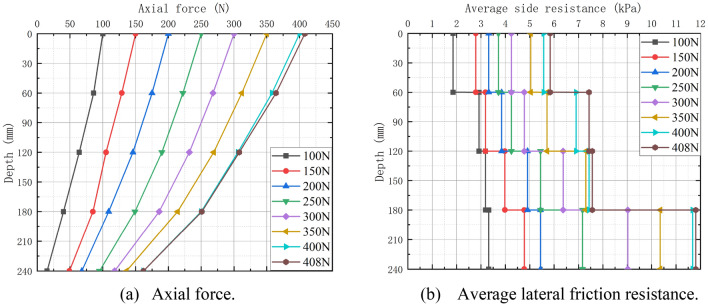
Axial force and average lateral friction resistance along the model pile.

The graphs of pile shaft force and average lateral frictional resistance are depicted in Fig. 7. In the early stage of force application (Fig. 7a), the pile shaft force is distributed roughly proportionally with depth, and the curve climbs in a similar manner. As the tension approaches its maximum load, the ratio between axial force and displacement changes rapidly in the last stage. Under the same load, it is discovered that the pile shaft force reduces with increasing depth; at the same depth, the shaft force increases with increasing load. The average lateral friction resistance of each part of the pile increased with the uplift load (Fig. 7b), and it increased with the depth under the same weight; the average lateral friction resistance reached its highest value at 408 N.

#### Analysis of load-bearing with different burial depths

**Figure 8:**
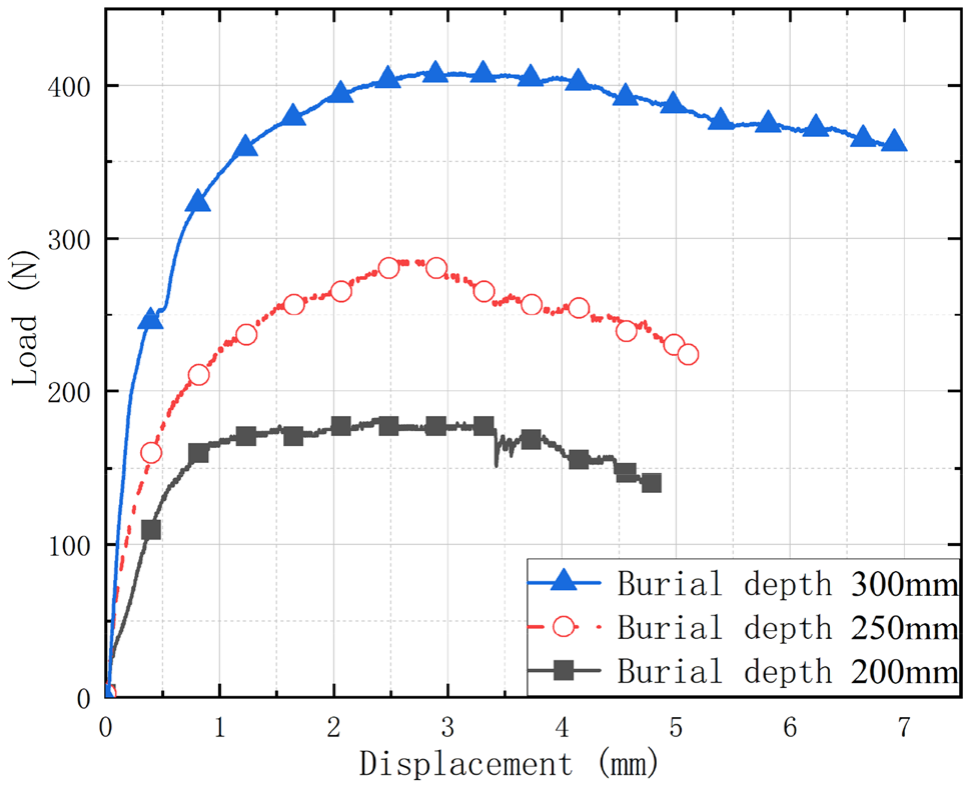
Pile top Q-s curves with different burial depths.

The *Q*-*s* curve at various burial depths was generated by combining the load applied by the universal machine and the recorded displacement data, as depicted in Fig. 8. During the initial phase of uplift, the pile top load rises abruptly, then declines gradually after reaching its peak, and finally reaches a more moderate and steady level. According to ASTM D3689-07^24^, the load corresponding to the starting point of the *Q-s* curve with an obvious steep drop is taken as the ultimate load, so the ultimate bearing capacity measured when the pile is buried 300 mm, 250 mm, and 200 mm is 408 N, 285 N, and 182 N, respectively, indicating that the pile foundation uplift bearing capacity will decrease as the burial depth decreases. To ensure the safety of the project and maximize the pullout resistance, the burial depth of the pile must be rationally established in engineering practice based on the condition of force.

**Figure 9:**
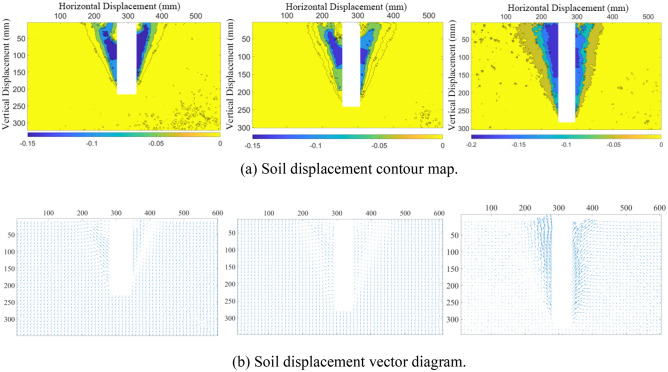
Soil displacement with different burial depths (200 mm, 250 mm, 300 mm).

Figure 9 illustrates the displacement clouds and vector diagrams for different burial depths. In Fig. 9a, the pile surface is plastered with sandpaper in order to increase the pile-soil interface frictional resistance. The frictional resistance is not the same at different burial depths, and the horizontal displacement of the perimeter soil of the engineering control pile with vast burial depths has a wide impact range, but in the end, they all exhibit an inverted cone damage shape. According to Fig. 9b, the soil arch effect at the top of the pile grows as the burial depth increases, and this effect can be observed as the surface soil layer rises.

**Figure 10:**
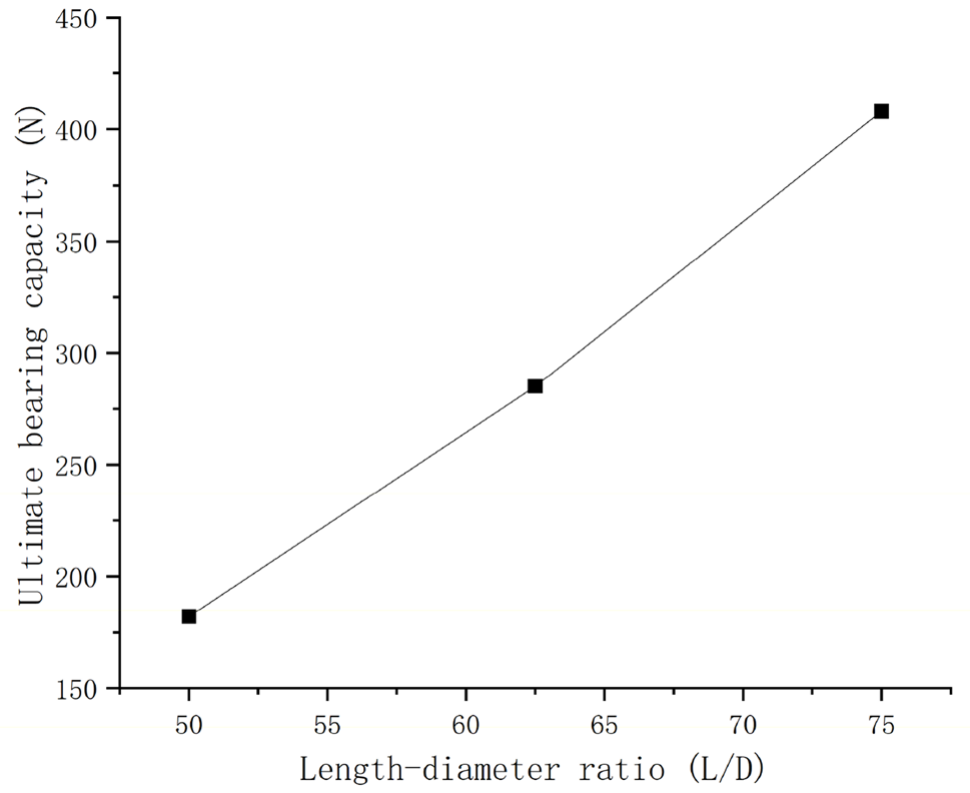
Relationship between Ultimate bearing capacity and length-diameter ratio.

Figure 10 shows the ultimate bearing capacity with different length-diameter ratio (L/D, 50, 62.5, and 75 respectively). The ultimate capacity increased with the increase of length-diameter ratio. For the general equal-section pile, the damage surface of surrounding soil mainly has the following damage patterns: cylindrical damage, inverted cone damage, and compound damage (i.e., cylindrical damage in the lower part and inverted cone damage in the upper part). As shown in Fig. 9a, the damage pattern appeared in this study can be identified as the second damage pattern mentioned above, which was caused by the increase of friction due to the pile was frictional. And the failure pattern of this test was consistent with previous study^22^.

### Analysis of numerical model results

#### Numerical model construction

**Figure 11:**
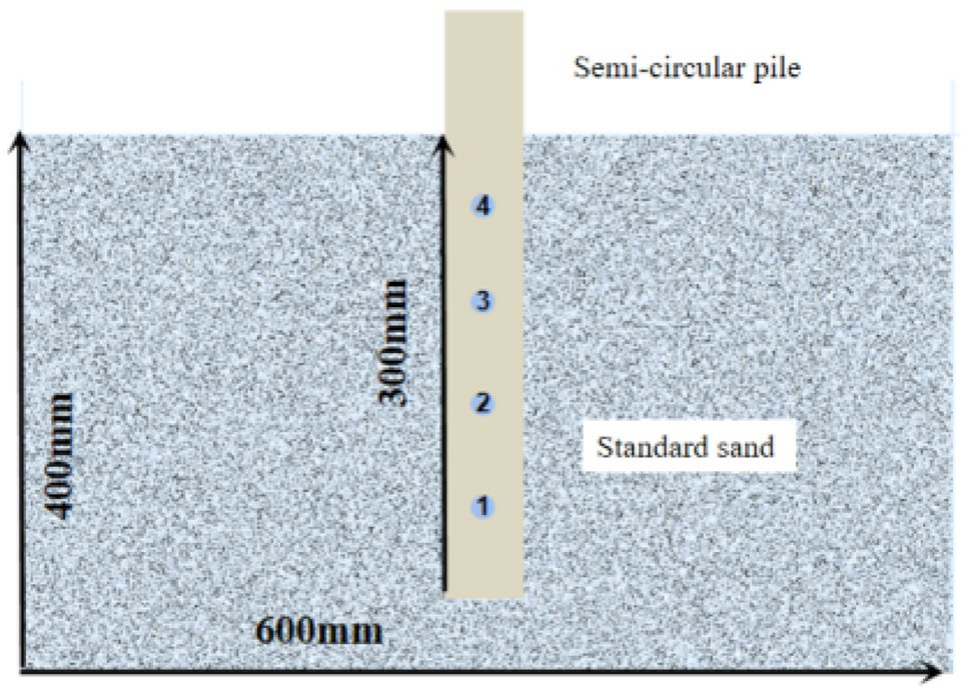
Setup of numerical model.

The numerical model test of the test pile with a 300 mm burial depth was conducted using DEM to investigate the micro-scale internal laws of the uplift process of the uplift-resistant pile and to validate it with an indoor model test. The particle density of the soil is 2650 kg/m^3^, and the micro view parameters are displayed in Table 1. Figure 11 depicts the numerical model diagram of the 300 mm-buried pile, which is consistent with the test condition. Four monitoring points for axial force were established 60 mm, 120 mm, 180 mm, and 240 mm from the base of the pile (locations 1, 2, 3, and 4).

**Table 1:** Parameters used in the numerical modeling.

Contact type	Soil-soil	Soil-pile	Pile-pile
Effective modulus (Pa)	8 × 10^6^	8 × 10^6^	5 × 10^7^
Stiffness ratio	2.0	1.0	2.65
Friction coefficient	0.3	0.5	0.5
Rolling friction coefficient	0.2	0.2	–
Parallel bond modulus (Pa)	–	–	1 × 10^9^
Parallel bond stiffness ratio	–	–	1.0
Parallel bond cohesion (Pa)	–	–	1 × 10^15^
Parallel friction angle (°)	–	–	20
Tensile strength (Pa)	–	–	1 × 10^15^

#### Validation of DEM model

**Figure 12:**
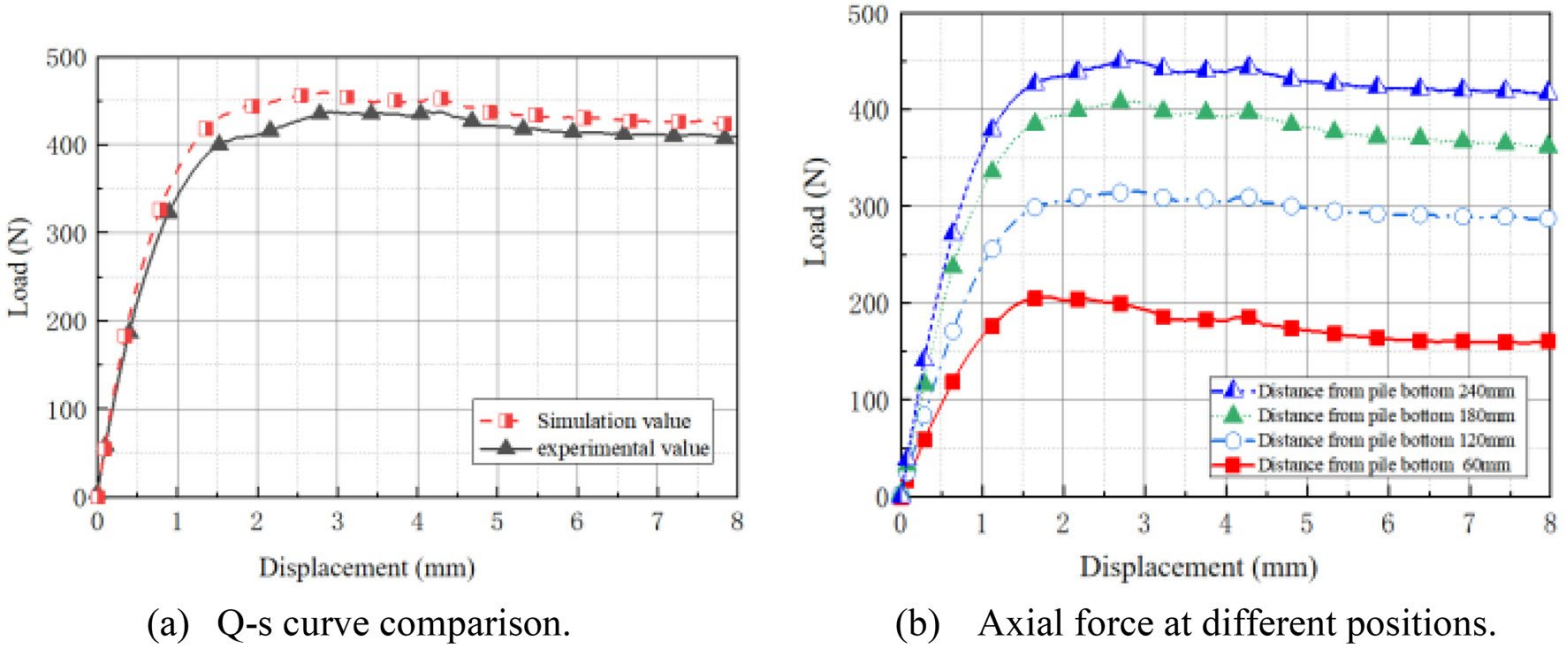
Pile loading results of DEM model.

During the uplift of the model pile, the bearing capacity and lateral frictional resistance are always in tandem, with the frictional resistance being somewhat less than the bearing capacity. When comparing the *Q*-*s* diagrams of test and simulation (Fig. 12a), it is discovered that the model pile reaches its ultimate state of bearing capacity at a displacement of approximately 3 mm, and that, although there is a small decrease with the growth of displacement, the final difference with the ultimate bearing capacity is not large, and the relative error between them is within 10%, confirming the accuracy of simulation. Additionally, the pile axial force increases as up-drawing displacement increases (Fig. 12b). 4 axial force is obviously superior to 3 axial force, 2 axial force, and 1 axial force. The fluctuations in axial force at four separate locations follow a similar pattern; only their magnitudes vary. When the displacement reaches the limit value, the resistance growth becomes slow.

**Figure 13:**
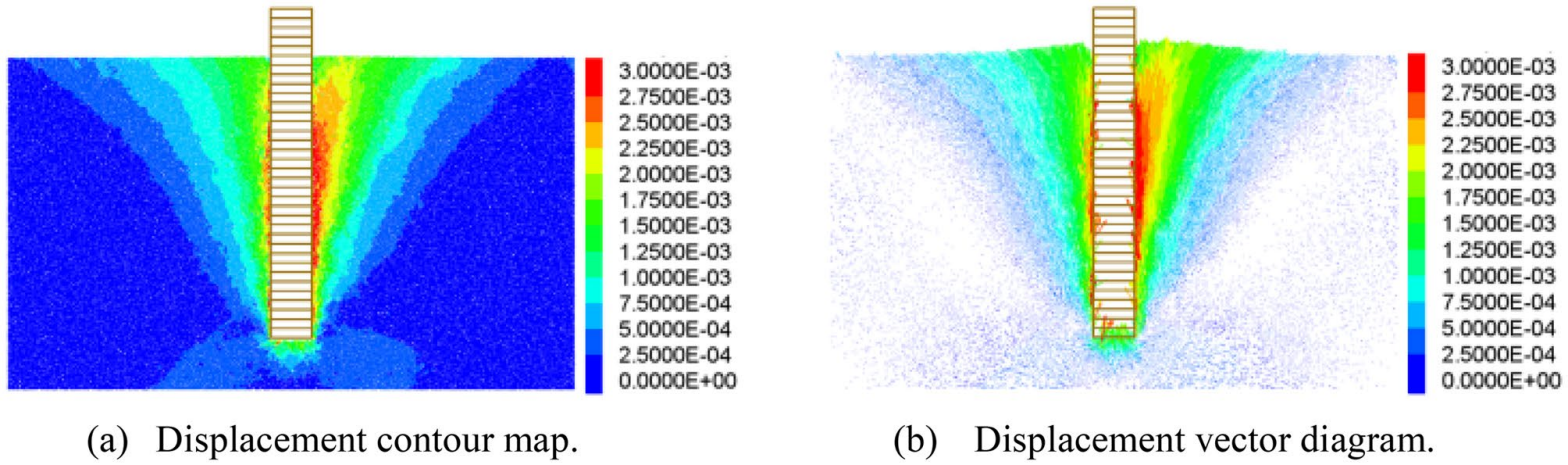
Soil displacement results of DEM model.

Figure 13 depicts the soil displacement results of the 300 mm-deep pile model. The cloud exhibits a V-shaped spread from the base of the pile to the top, and the displacement of soil particles near the pile has the greatest impact, whereas the rest of the soil is less stressed. The top of the pile is accompanied by a similar ground rise in the vector diagram. This is consistent with the soil displacement cloud and vector diagram (Figs. 5 and 6) observed by DIC during the test, indicating that the test was conducted correctly.

#### Analysis of force chain

**Figure 14:**
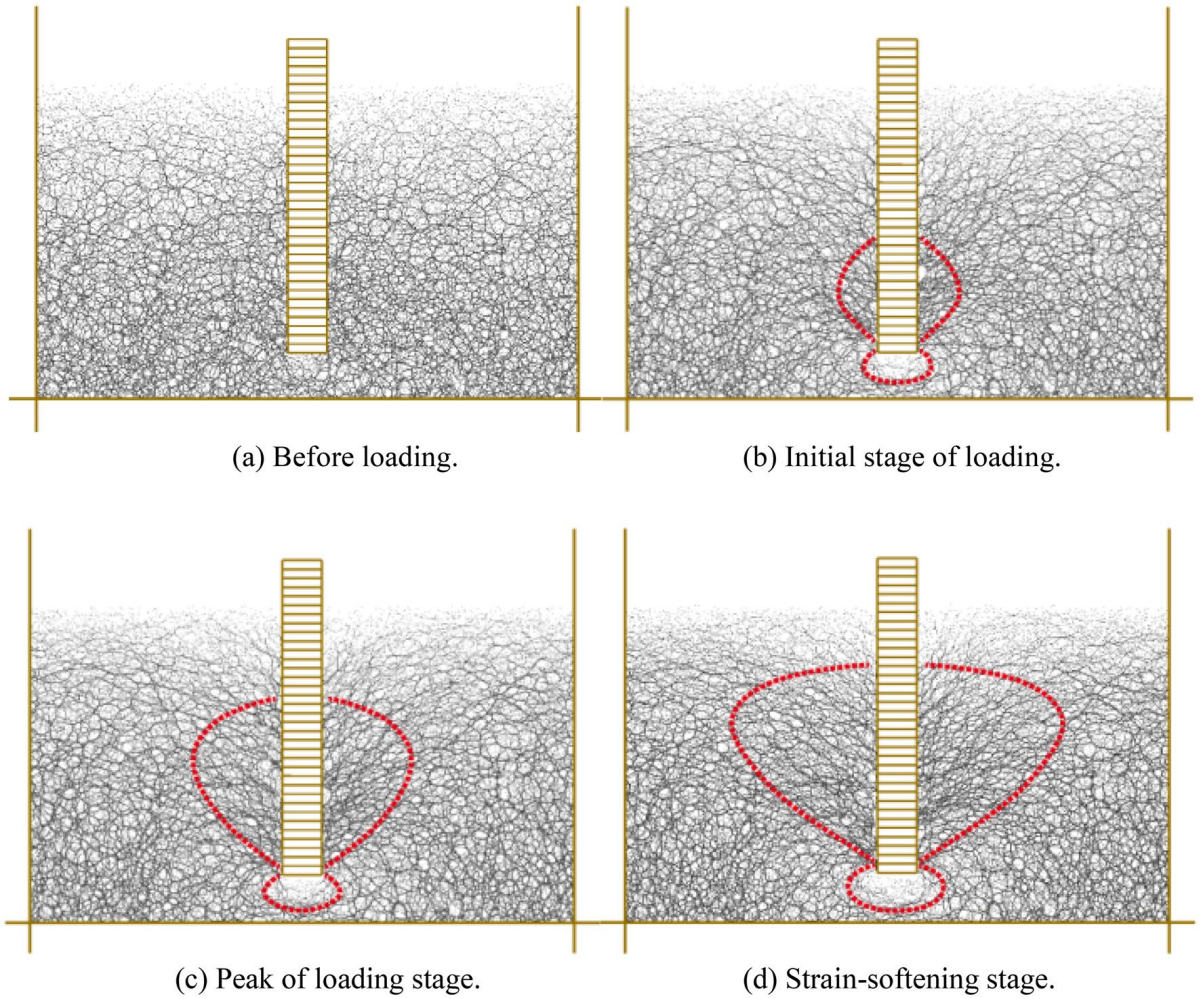
Force chain distribution at different stages of loading.

The chain-like structure generated by force transfer between particles in discrete media is known as a force chain, and the distribution of force chains reflects the load transfer behavior of discontinuous media. Figure 14 depicts a comparison of the distribution laws of contact force chains during four distinct phases of the pile extraction procedure. The thicker the force chain, the greater the contact force. Before loading, the distribution of force chain became dense from top to bottom because of the gravity. When loading started, elliptical force chain cavities began to emerge at the bottom of the pile, and the force chain concentration distribution around the bottom surface of the pile was arch-shaped. Throughout the peak of loading stage, the force chain concentration zone expands more outward. When it comes to strain-softening stage, the side of the pile grew outward from the bottom to the top of the pile, forming a convex arch force chain concentration area. Comparing the distribution of force chains in the four stages reveals that the density and spread of force chains gradually increase and expand with the increase of lateral friction resistance; the force chains on both sides of the bottom of the pile gather rapidly, causing the lateral friction resistance to increase first and then gradually expand to form a stable force chain network.

#### Analysis of Stress field and anisotropy

The pile extraction is inevitably accompanied by stress redistribution in the foundation soil, and a 36 × 21 measure spheres matrix is arranged in the model to obtain the stress distribution in the soil for further analysis of the pile-soil load transfer mechanism. The principal stress direction vector is used to characterize the principal stress variation properties in the soil. The principal stress values and their direction angles are calculated by Eq. (1):

1 $$\left. \begin{aligned} & \theta = \frac{1}{2}\arctan \left( {\frac{{ - 2\tau_{xy} }}{{\sigma_{xx} - \sigma_{yy} }}} \right) \hfill \\ & \sigma_{1,3} = \frac{{\sigma_{xx} + \sigma_{yy} }}{2} \pm \sqrt {\frac{{\left( {\sigma_{xx} - \sigma_{yy} } \right)^{2} }}{2} + \tau_{xy}^{2} } \hfill \\ \end{aligned} \right\}$$

where *σ*_*xx*_, *σ*_*yy*_, *τ*_*xy*_ is the positive and tangential stresses in the x and y directions of the soil recorded in the measurement circle; *σ*_1,3_ is the size principal stress; *θ* is the direction angle of the large principal stress relative to the initial direction (vertical direction).

**Figure 15:**
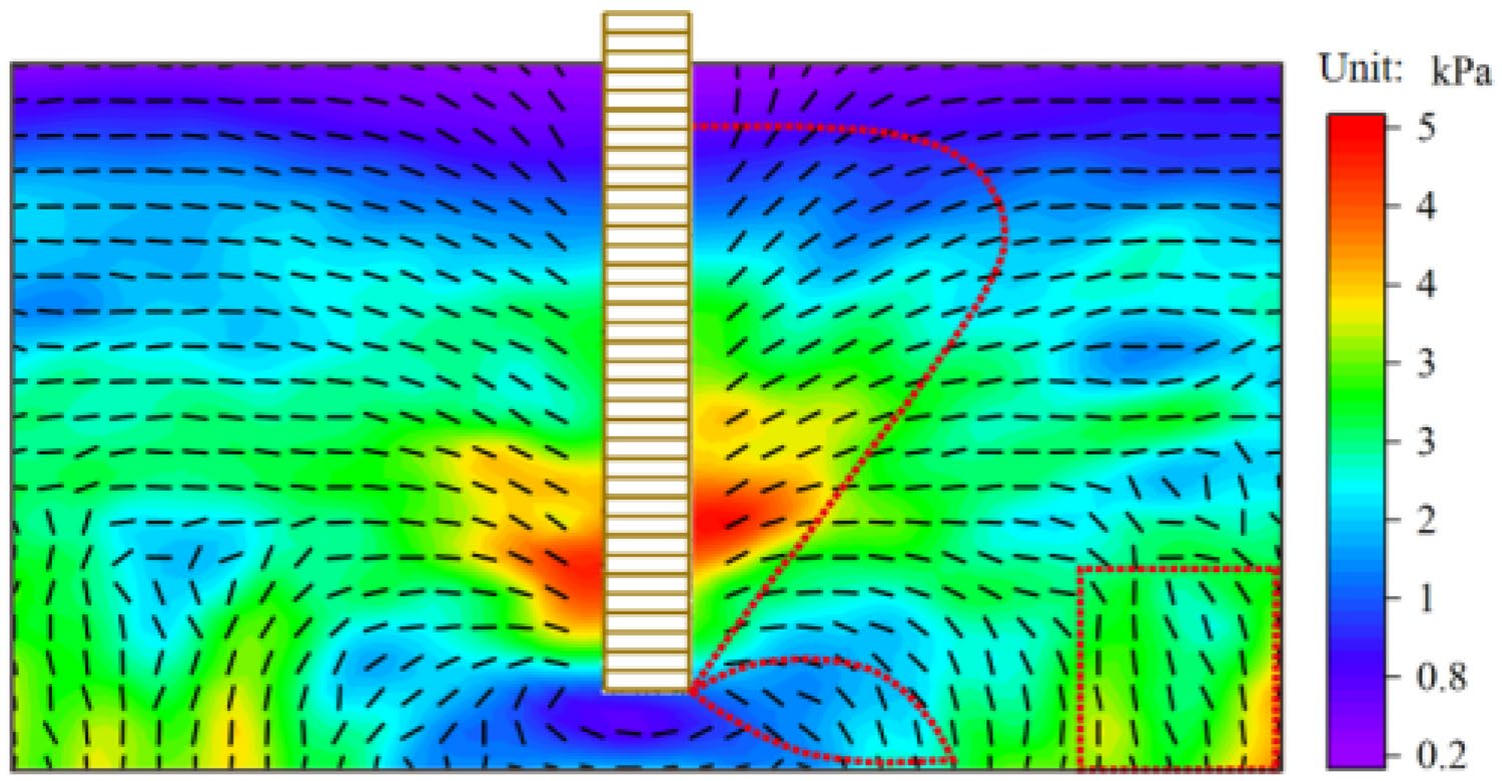
Large principal stress and direction vector diagram.

The strain-softening phase is the most severe phase of internal soil degradation. During this stage, Fig. 15 depicts the huge principal stress field and its direction vector in the foundation soil. From the top of the pile to the bottom of the pile, the value of the principal stress in the soil around the pile first increases sharply to its maximum value and then decreases sharply, with the highest value of the principal stress indicating that this portion of the pile is currently bearing most of the soil pressure within the soil. Under the ultimate load, the pile lateral frictional resistance grows throughout the depth and then declines quickly, consistent with the model test pile lateral frictional resistance research findings. The direction zone of the large principal stress vector of the foundation soil is comparable to the distribution range of the force chain. The large principal stress vector is deflected to the side and bottom of the pile, and the respective ranges of the direction zone are convex arch and lobe. The direction of the soil near the bottom of both sides of the boundary is extremely minor and essentially unaffected by the pile extraction, while the stress value is raised under the combined action of gravity and load transfer from the pile side compared to other areas with minimal direction. When the soil particles have developed a somewhat stable structure, the direction of the large principal stress vector in the foundation soil and the growth of the force chain create internal force redistribution.

When the stress is redistributed, the soil particles will spontaneously adjust the inter-particle contact state to adapt to the changing stress field, Rothenburg and Bathurst^25^ used the inter-particle contact distribution function to analyze the contact configuration of particles, which can quantify the variation of inter-particle contact distribution within the foundation, see Eq. (2)

2 $$E(\theta ) = \frac{1}{2\pi }\left[ {1 + a_{{\text{n}}} \cos 2\left( {\theta - \theta_{{\text{n}}} } \right)} \right]$$

where *E*(*θ*) is the density function of the contact normal distribution; *θ*_*n*_ is the main direction of the contact normal anisotropy (angle with the horizontal line); *a*_n_ is the Fourier fit coefficient, characterizing the complexity of the anisotropy.

**Figure 16:**
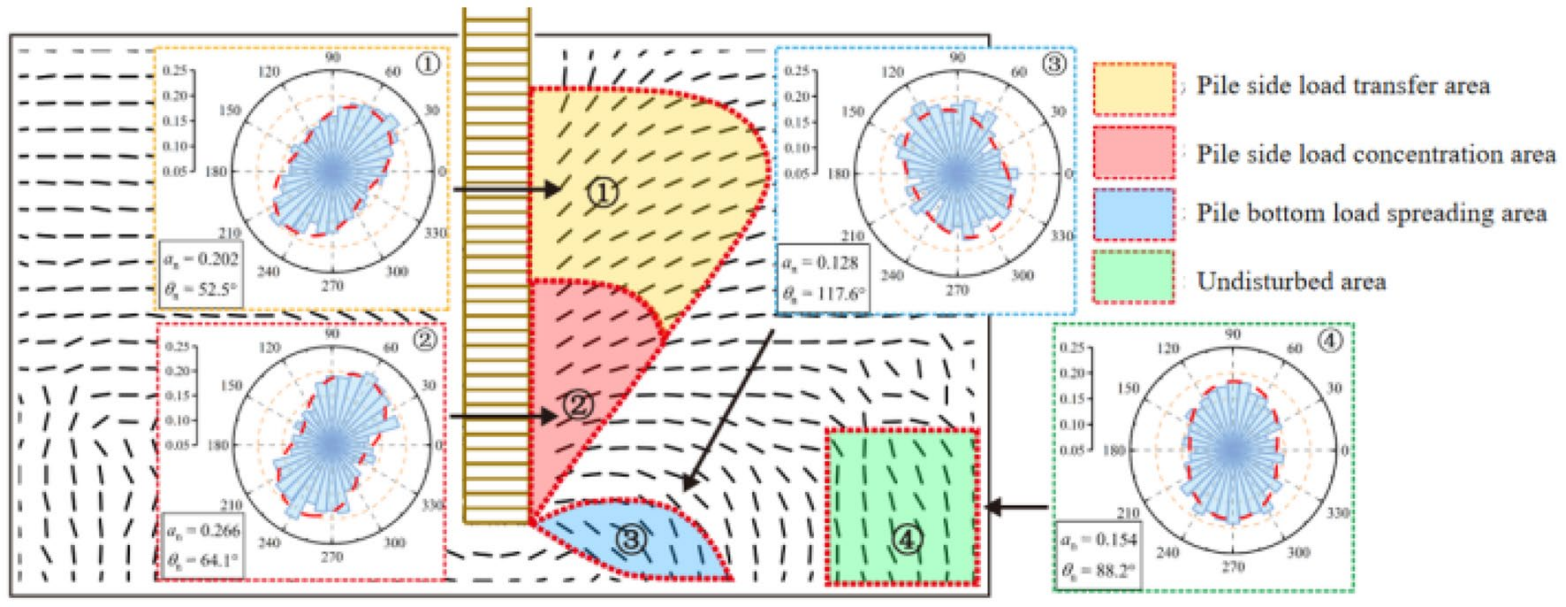
Distribution of the contact normal direction.

According to the values of the foundation soil large primary stresses and vector directions, the areas were numbered and subdivided^26^. The principal direction of the contact normal direction of the particle system was counted as a statistical interval of 10° and fitted with a Fourier function to generate the analysis diagram of the contact normal configuration in the peak of loading stage, as shown in Fig. 16. The proportion rose slice reflects the ratio of contacts in that direction to the average number of contacts along the entire direction, and the primary direction of the normal configuration of the particle contacts in each region has a high similarity to the stress direction (Fig. 16). The coefficients of anisotropy of the soil in each region (1 to 4) are 0.202, 0.262, 0.128, and 0.154, respectively, and the degree of anisotropy of the particles in the region of pile lateral load concentration is the greatest. The greater the degree of anisotropy, the more concentrated the contact force necessary to withstand an external load. The closer the condition of inter-particle contact, the greater the squeezing effect on the particles and the greater their capacity to transfer load. Under the double extrusion of lateral earth pressure and pile lateral frictional resistance, the interparticle contact state is tighter; while the anisotropy coefficient in the pile bottom load spreading zone is the lowest, most of the earth pressure in the foundation is shared by the pile, and only a small amount of external load and gravity perturbation occurs, the interparticle contact state is looser. At the juncture of the two zones, the pile lateral frictional resistance diminishes drastically. Comparing the transfer zone and the concentration zone, the main direction of the contact normal is found to be closer to the vertical direction in the concentration zone, along with a higher degree of anisotropy. This indicates that the particles in the concentration zone have a greater capacity to withstand the vertical parting pulling load.

## Conclusions

In this study, lab tests and numerical analysis are used to investigate semicircular thin-walled piles subjected to vertical uplift loads in sandy soils, and the following conclusions can be obtained:

1. Under the uplift load, the pile primarily undergoes four stages: initial stage of loading, strain-hardening stage, peak of loading stage, and strain-softening stage. With stress increasing, the soil surrounding the pile exhibits inverted conical damage, and a surface soil arch phenomenon becomes evident. During the uplift process, the axial force of the pile under the same load decreases in the depth direction and increases as the uplift load at the same depth increases. Simultaneously, the pile lateral frictional resistance develops in the depth direction (60 mm from the pile bottom) and with the uplift load. The ultimate capacity increased with the increase of length-diameter ratio (L/D).
2. During the pile uplift test, the DIC image quantifies the disruption process of the surrounding soil. The soil disturbance displacement generated by the uplift displacement at pile depths of 300 mm, 250 mm, and 200 mm exhibits a V-shaped pattern around the pile, and the effect range of the soil surrounding the pile grows as the pile depth increases. Soil particles surrounding the pile appear arch phenomena at the surface. Currently, the distribution of soil displacement vectors is the densest due to the greatest disturbance force.
3. The lateral frictional resistance of the pile modeled by DEM is always slightly less than the ultimate bearing capacity. The axial force at four selected places along the pile follows the same pattern as the final bearing capacity curve, except for the magnitude. The simulated bearing capacity curve is identical to the pile load–displacement curve of the test; the simulated soil displacement cloud and vector diagram around the pile are identical to the overall damage trend measured by DIC in the test, and the soil protrudes from the top surface of the pile. Therefore, the DEM simulation and the test can be mutually validated, demonstrating the validity and viability of the study.
4. From the micro perspective, the force chain diagram of the DEM simulation helps depict the force transfer process of pile lateral friction resistance to surrounding soil particles. The analysis of the large principal stress field and its direction vector in the foundation soil during the peak of loading stage reveals that the large principal stress reaches its maximum at a certain distance from the pile end, indicating that the pile lateral frictional resistance first increases and then decreases sharply along the depth. The direction vector diagram is partitioned and quantified. Compared with the transfer area, the concentrated area has a higher degree of anisotropy, and the major direction of the contact normal is also closer to the vertical direction, which means the particle has a stronger ability to resist the vertical pullout load.

## Data Availability

The data used in this work can be made available upon reasonable request to the corresponding author.
